# Tuberculosis at Home and Abroad

**Published:** 1919-09-27

**Authors:** 


### The Hospital

The Workers' Newspaper of Administrative Medicine and Institutional Life, Administration, National Insurance and Health.

No. 1738, Vol. LXVI. SATURDAY, SEPTEMBER 27, 1919. PRICE TWOPENCE

### TUBERCULOSIS AT HOME AND ABROAD.

This war was hardly likely to fail of war's usual sequelæ, the chief of which are three: Byron's "sister-evil" taxation, and famine, and pestilence. The worst pestilence has been tuberculosis, and that again has been most severe where privation is hardest, namely, in Austria and Germany, where hospitals have no soap and no bed linen. And the question arises, can this foreign war tuberculosis, extreme in character, be brought over to this country, where the disease, though much above normal in extent, is still by comparison moderate? Many diseases have been imported from war zones, and as these are still plentiful, should we look for other immigrant sickness? There have been several little outbursts of smallpox up and down the country, the latest at Manchester. There has been rabies. There has been a great deal of itch. Influenza probably came from the Continent: at any rate it was there before it was here, here before it was in Australia. In most of the smallpox outbreaks a returned soldier seems to have been the first patient, and similarly with the scabies. On the other hand, the malarial soldier from the East, Salonika or Mesopotamia, as the case may be, has been no danger to others. His ague fits did not cease with the change of climate, but nobody else among his family or friends got them. They did not have to take quinine, as the others had to take sulphur baths or be isolated with their pustules. Now what about the tuberculous soldier—is he like the one with smallpox or like the one with ague?

Well, up to very lately most of our cognoscenti would have answered that he decidedly resembled more the one with smallpox. Our contagionist friends, leaning hard, probably over hard, upon what were in reality rather crude laboratory experiments, with little real resemblance to actual human conditions of life, were accustomed to preach vehemently infection as the great cause in the spread of tuberculosis. The human bacillus was caught pretty directly from some case of human tuberculosis, and the bovine one from tuberculous meat or dairy products. Koch was wrong in minimising the latter risk, but quite right in advocating the strict isolation of all advanced consumptives. Look after each new case as it cropped up, prevent it from being a source of infection, take care there was no death in the pot or in the milk-jug, and tuberculosis would fade away, if not as quickly as Malta fever did, at any rate within the next two or three decades. The first shaking this position had was the demonstration that the bovine bacillus, though not as innocuous to mankind as Koch had said, was still of fairly minor importance. Even surgical tubercle, the tuberculosis pre-eminently of children, the chief consumers of milk, was traceable much more often to the human type of germ than to the bovine one, while the enterogenous origin of consumption became discredited. The publicists who had preached death in the pot, to the admiration of our sensational lay press and the dismay of farmers, did not retract their statements—how rarely that is done!—but switched themselves on to another line of talk. Howbeit other accepted doctrines seem to be now in danger, for the effect of the war has not been at all in accordance with orthodox expectation. *The war brought an increase of tuberculosis out of all proportion to the increase of the chances of infection.* The voluntary or conscripted soldier who became tuberculous had run no extra risk of infection; indeed from his open-air drill and association with physically selected individuals he had probably run less risk. But the shake to his constitution which hardship and a radical change in mode of life caused depressed his physiological fitness and thereby his natural power of resistance, his *vis medicatrix*. Much the same with the munition girl. In the well ventilated and sanitated factory she had probably better air to breathe than in the kitchen or back parlour she came from; she too mixed with people who had been medically examined. But her suddenly started long grind of work, amongst machinery that needed to be carefully watched, told upon her general health too. Nowhere was there sudden increase of infection; everywhere there was sudden increase of disease. Diminution of food, without war, cannot possibly mean higher chances of tuberculous infection. And yet the neutrals, deprived of food by the combatants, suffered more with tuberculosis than they had done before. Very obviously, in the teaching current pre-war concerning the pathogeny of pulmonary tubercle, the doctrine of *latency* had been underrated. We know that a tuberculosis lesion may neither worsen nor quite heal, but remain like a sleeping dog for years and years, biding its time until constitutional resistance becomes depressed; and we also know that minute quiescent lesions are common enough in apparently healthy persons. So at a time when constitutional depression is widespread, tuberculosis becomes widespread too. These are from a practical point of view the important factors—constitutional resistance and the almost inevitable latent focus. Direct infection, contacts, isolation, are, save in the case of youngish children, much less important than most of us thought them in 1914. Already there are signs of some eagerness to vote for this amendment, which we do not doubt will be duly carried. After that there is another in store, relating to the still overlooked factor of predisposition. But that is prophecy.

Meanwhile, returning to our muttons, we deduce that the terrible war-tuberculosis, still as active, apparently, as we see war-lying, calumny by suppression, tyranny, hypocrisy—all the world-old manifestations of *homo homini lupus*—is not likely to come to these islands. And it may burn itself out where it is not very slowly, for they say to get tuberculosis in Germany or Austria is equivalent to sentence of death, the disease being virulent and proper support unobtainable. Besides, there is the possibility of German chemo-therapy finding the specific remedy, of which it may even now be on the track: the testing of chemo-therapeutical compounds by Much's "partigen" reactions seems by their tuberculosis journals to be attracting a good deal of attention, although, so often in the past have hopes been raised without much result in every civilised country by some worker or other, it would not have called for mention from the practical point of view had it not been for the achievement of the Finkler chemo-therapy in the case of lupus, lately alluded to in these columns. But that is all as it may be. What we are here concerned to emphasize is this point merely: *clinical tuberculosis is not a miasma, but a blight that fastens on the unhealthy plant.*

